# High degree of circular polarization in WS_2_ spiral nanostructures induced by broken symmetry

**DOI:** 10.1038/s41598-019-39246-7

**Published:** 2019-02-26

**Authors:** Prahalad Kanti Barman, Prasad V. Sarma, M. M. Shaijumon, R. N. Kini

**Affiliations:** School of Physics, Indian Institute of Science Education and Research Thiruvananthapuram (IISER-TVM), Maruthamala P.O., Vithura, Thiruvananthapuram, Kerala 695551 India

## Abstract

We present helicity resolved photoluminescence (PL) measurements of WS_2_ spiral (SPI) nanostructures. We show that very high degree of circular polarization (DCP) (~94 ± 4%) is obtained from multilayer SPI samples at room temperature upon excitation with a circularly polarized laser at a wavelength near-resonant with the A-exciton (633 nm). TEM analysis showed that these SPI nanostructures have AB stacking in which the inversion symmetry is broken, and hence this leads to very high DCP. Comparison with PL from monolayer and bi-layer WS_2_ samples, along with polarization resolved PL studies provide evidence for suppression of interlayer/intravalley scattering in the multilayer SPI samples.

## Introduction

Layered two-dimensional (2D) transition-metal dichalcogenides (TMDCs) have received a great deal of attention recently because of their interesting layer-dependent properties, which are different from that of the bulk. For example, several of these TMDCs have an indirect bandgap in the bulk form while as a monolayer (ML) they exhibit a direct band gap^1–3^. The band-edge of these MLs is located at energy degenerate valleys at the corners of the hexagonal Brillouin zone. Because of the lack of inversion symmetry in the ML, the inter-band transitions in the vicinity of the *K* (*K*′) point couple exclusively to the right (left) handed circularly polarized light^4–6^. This opens up the possibility of using TMDCs for valley based electronic and optoelectronic applications^7,8^. Valley polarization was first reported in MLs of MoS_2_ where excitation with circularly polarized light, resonant with the A-exciton (AX) energy yielded circularly polarized luminescence^9,10^. Because of inter-valley scattering, the degree of valley polarization was negligible at room temperature. Also, since the inversion symmetry is restored in bilayers (BLs), only a small degree of valley polarization was observed in BL MoS_2_^11,12^. WS_2_ is another TMDC in which the control of the valley polarization could be realised. Interestingly, unlike its sister compound, a high value of valley polarization (~95%) was reported in BL WS_2_ at low temperatures (<120 K) and later it was shown that it is possible to achieve a high degree of valley polarization (~80%) in BLs even at room temperature by using near-resonant excitation^13,14^. Valley polarization is expected to be absent in BL TMDCs since the inversion symmetry is restored and hence observation of high valley polarization in WS_2_ BL was surprising. Strong circularly polarized trion luminescence (~60%) has been observed, in BL WSe_2_ at ~30 K with above bandgap excitation, but it was attributed to exciton spin polarization and “spin-layer locking effect” and not to valley polarization^15^. The coupling between spin-valley and layer pseudospin is much stronger than interlayer hopping in TMDCs because of the strong spin-orbit interaction of the *d*-orbitals of the heavy metal atoms^16^. Hence it was proposed that the spin index becomes locked with the valley index at the band edges and because of the interplay with the layer pseudospin, the spin orientation is locked to the layer pseudospin in each valley in BL TMDCs. It was argued that this leads to highly circularly polarized emission in inversion symmetric BLs of TMDCs. Later, using first-principles calculations, it was shown that the spin polarization localized on the individual layers in a BL has the same magnitude but opposite direction and hence is “hidden”^17^. This “hidden” spin polarization can give rise to strong circularly polarized emission even in centrosymmetric BLs as well as multilayers. Here, we show that a high degree of circular polarization (DCP) at room temperature is obtained (~94 ± 4%) in multilayer WS_2_ spiral (SPI) nanostructures. The SPI nanostructures grow via a screw dislocation-driven mechanism and consist of a single layer extending onto itself, in a spiral fashion, finally resulting in a multilayer triangular pyramidal structure. The stacking configuration of the SPI nanostructure is AB^18–22^, in which the inversion symmetry is explicitly broken^23^. We show that for AB stacking SPI nanostructures, because of the broken inversion symmetry, the DCP is high. This opens up the possibility of using a wide range of TMDC nanostructures for novel quantum devices since the interplay between the spin, valley and layer pseudospin can be controlled by stacking sequence, magnetic, optical and electrical means in these materials.

## The Results and Discussion

**Figure 1:**
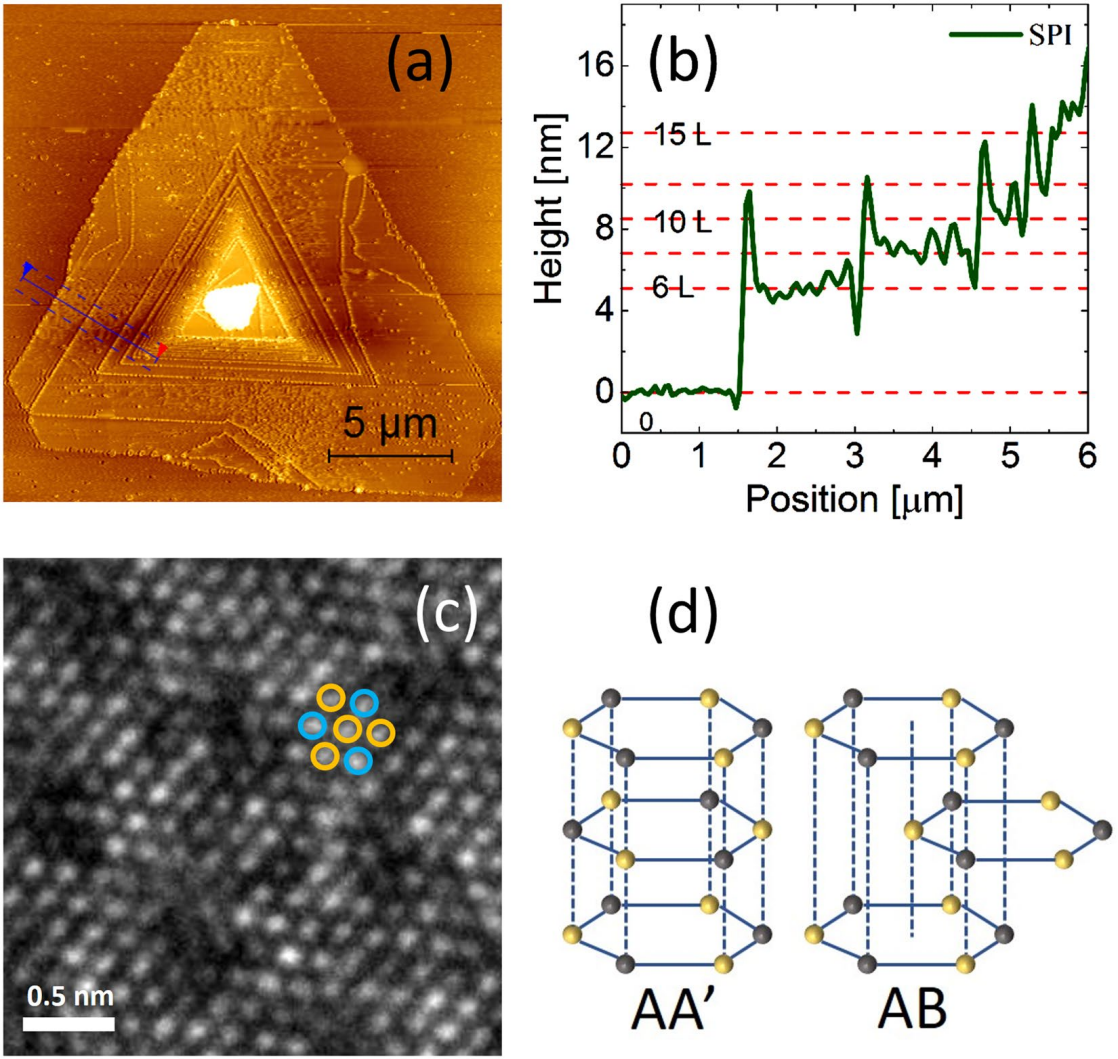
(**a**) AFM image and (**b**) the height profile of SPI WS_2_ nanostructure along the marked blue line in (**a**). (**c**) The HRTEM image of SPI WS_2_ nanostructure. (**d**) A schematic illustration of AA′ and AB stacking of WS_2_ layers_._

ML, BL, and SPI WS_2_ nanostructures were grown by the CVD technique on Si/SiO_2_ (290 nm SiO_2_) by direct sulphurization of WO_3_ precursor as reported earlier^24^. The morphology of the SPI nanostructures was confirmed using atomic force microscopy (AFM) as shown in Fig. 1(a) and Supplementary Fig. S1. The cross-section shows plateaus with a step of size ~0.85 nm which matches with the thickness of a single atomic layer of WS_2_ as shown in Fig. 1(b). To understand the microscopic structure of the SPI nanostructures, we transferred these on to a transmission electron microscopy (TEM) grid and did TEM measurements. Figure 1(c) shows a TEM image of one typical WS_2_ SPI nanostructure. In the case of WS_2_, two stacking orders are possible, referred to as AA′ and AB as illustrated in Fig. 1(d). For AA′ stacking the in-plane W-S bonds are opposite for the two adjacent layers, the layers eclipse each other and belong to the centrosymmetric *D*^1^_*3d*_ group. However, in the case of AB stacked order, the in-plane W-S bonds point in the same direction in the adjacent layers, the layers are staggered with respect to each other and belong to the non-centrosymmetric *D*^1^_*3h*_ group. Both AB and AA′ are stable configurations of WS_2_. The TEM analysis shows that the WS_2_ SPI nanostructures have AB stacking, similar to the case of SPI MoS_2_ nanostructures^21^.

**Figure 2:**
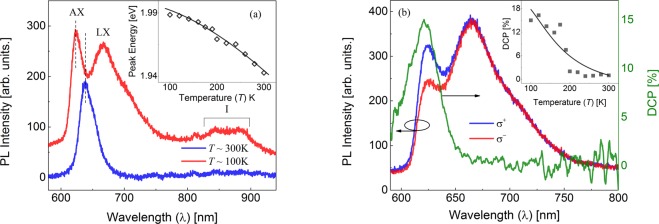
(**a**) PL spectra of WS_2_ SPI nanostructure obtained at ~100 K and ~300 K using non-resonant (532 nm) linear excitation showing the A-exciton (AX), localized exciton (LX) and indirect excitonic (I) features. The data has been shifted vertically for clarity. The inset shows the temperature dependence of the AX peak. The solid line is a fit to the Varshni equation. (**b**) Helicity resolved PL spectra obtained using right-handed circularly (*σ*^+^) polarized 532 nm excitation and the corresponding DCP at ~100 K. The inset shows the DCP as a function of temperature. The solid line is a fit to the experimental data as described in the main text.

Figure 2(a) shows the comparison of the room temperature and low temperature (~100 K) PL spectra obtained by exciting a WS_2_ SPI sample with a linearly polarized 532 nm laser. The dominant peak near ~637 nm is due to the recombination of neutral excitons (AXs)^25,26^. The PL spectra taken at 100 K shows an additional peak near ~666 nm which is due to the localized excitons (LXs)^26^. Additionally, a broad feature near ~850–900 nm is also observed possibly due to indirect bandgap transitions (I)^13,21^. Compared to the WS_2_ ML and BL samples (see Supplementary Fig. S2), the SPI structure has the lowest PL intensity when measured under similar experimental conditions, which further confirms that the SPI structure consists of a multilayer structure^21,24^. The temperature dependent PL measurements show that the PL peak blue shifts with a decrease in temperature. As shown in the inset of Fig. 2(a), the temperature dependence of the PL peak energy can be fit well with the empirical Varshni equation. The fit to the Varshni equation for the ML and BL samples is shown in the Supplementary Fig. S2 which agrees with earlier reports^27,28^.

To study the spin-valley polarization in these nanostructures, we excited the samples with circularly polarized light and analyzed the polarization state of the emitted PL. We define the DCP as,

$${P}_{circ}( \% )=\frac{{I}_{{\sigma }^{+}}-{I}_{{\sigma }^{-}}}{{I}_{{\sigma }^{+}}+{I}_{{\sigma }^{-}}}\times 100$$

Where ${I}_{{\sigma }^{+}}({I}_{{\sigma }^{-}})$ is the intensity of the right (left)-handed circularly polarized component of the PL.

### Non-resonant circularly polarized excitation

Figure 2(b) shows the intensities of the left and right-handed circularly polarized components of the PL along with the DCP for a WS_2_ SPI sample at ~100 K, obtained using right-handed circularly polarized (*σ*^+^), 532 nm excitation. As one can see from Fig. 2(b), ~15% DCP is observed near the AX peak in the WS_2_ SPI sample. The degree of circular polarization decreases for wavelengths away from the AX peak. It is observed that for LX the degree of circular polarization is zero, which is similar to the case of MoS_2_^12,13^. We observed negligible DCP in the WS_2_ SPI structure at room temperature under non-resonant (532 nm) excitation. The intensities of the left and right-handed circularly polarized components of PL along with the DCP for a WS_2_ ML and BL samples at ~100 K, obtained using non-resonant excitation is shown in Supplementary Fig. S3. DCP of ~29% and ~25% were obtained at ~100 K for ML and BL samples respectively, which agrees with the reported values^13,14,29^. The inset of Fig. 2(b) shows the temperature dependence of the DCP in the WS_2_ SPI sample. The DCP can be expressed in terms of the exciton lifetime, *τ*_*r*_ and the intervalley scattering time, *τ*_*v*_ as ${P}_{{circ}}( \% )=\frac{1}{1+2\,\frac{{\tau }_{r}}{{\tau }_{v}}}$^28^. It has been shown that *τ*_*r*_ varies linearly with temperature^28^. The intervalley scattering time varies inversely with the phonon population, ${\tau }_{v}\propto \exp ({E}_{k}/{k}_{B}T-1)$, where *E*_*K*_ is the LA phonon energy near the *K*-points^10,28^. We have fit the data taking into account these two dependencies and the solid line shows the fit assuming an LA phonon energy of ~22 meV^30^. Similarly, as shown in Supplementary Fig. S3, the temperature dependence of DCP in ML and BL can also be fit taking into consideration LA phonon scattering. This confirms that the LA phonon scattering is the dominant mechanism responsible for the depolarization of the circularly polarized emission at elevated temperatures.

### Near-resonant circularly polarized excitation

**Figure 3:**
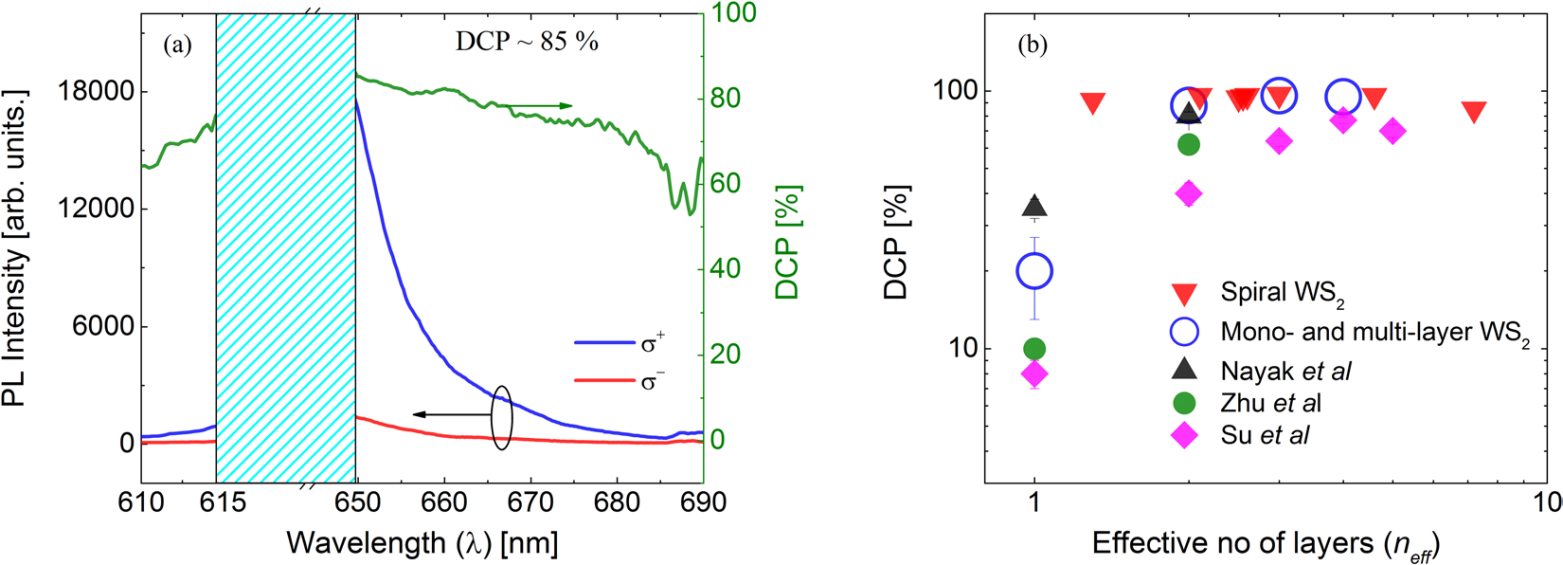
(**a**) Helicity resolved PL spectra obtained by using right-handed circularly (*σ*^+^) polarized near resonant (633 nm) excitation of twisted WS_2_ SPI structure and the corresponding DCP at room temperature (~300 K). The shaded region represents the stop band of the notch filter used to block the laser light from reaching the detector. (**b**) DCP at room temperature obtained for WS_2_ layers with different effective thickness using 633 nm excitation. For comparison, data from refs^14,31,13^ is also included.

We now turn our attention to the polarization resolved PL measurements on WS_2_ samples, done using a laser with wavelength (633 nm) near-resonant with the AX transition. Figure 3(a) shows the intensities of the left-handed circularly (*σ*^−^) and right-handed circularly (*σ*^+^) polarized components of the PL along with the DCP obtained with right-handed circularly polarized (*σ*^+^) 633 nm excitation of a WS_2_ SPI sample at room temperature. The wavelength range between 614 nm and 650 nm (shaded region) represent the stop band of the notch filter which was used to block the excitation laser line reaching the detector. The intensity of the LX is very weak compared to the non-resonant excitation case. As can be seen from Fig. 3(a), a very high degree of circular polarization (~85% measured at ~650 nm position) is obtained near the AX peak with right-handed circularly (*σ*^+^) polarized excitation. Similar value for DCP (~80%) is obtained for left-handed circularly (*σ*^−^)polarized excitation, as shown in Supplementary Fig. S4. We have done DCP measurements on a set of SPI WS_2_ structures (as shown in Supplementary Fig. S5 and have observed DCP in the range ~85–97% (average value: ~94%, standard deviation: ~4%), as shown in Fig. 3(b). DCP has been measured on a set of WS_2_ ML samples with the near-resonant excitation. In contrast to SPI structures, the DCP in WS_2_ ML is low at room temperature, even with near-resonant excitation condition (see Supplementary Fig. S6 (a)). For WS_2_ MLs, DCP values in the range of 13–34% was obtained, which is quite consistent with the previously reported values^29^. As shown in Supplementary Fig. S6(b), BL also shows a very high degree of circular polarization (~88%) with near-resonant excitation conditions, even at room temperature^14^.

Figure 3(b) shows the DCP obtained at room temperature for several WS_2_ SPI samples with varying layer thickness, as well as 1–4 layer-thick WS_2_ flakes along with the DCP values reported in the literature^13,14,31^. In the case of SPI samples, since the size of the laser spot (~5 µm-diameter) is bigger than the size of the plateaus, the laser excites several layers with different thicknesses at the same time. Hence using the PL intensity map of the samples, we arrived at an effective layer number by taking a weighted average of the number of layers over the PL intensity. If *I*_*n*_ is the PL intensity near the mid-point of the layer with thickness *n* layers and width *d*_*n*_ (See Supplementary Fig. S7), then the effective number of layers is obtained as: ${n}_{eff}=\frac{{\sum }_{n}n{I}_{n}{d}_{n}}{{\sum }_{n}{I}_{n}{d}_{n}}$. The DCP for the SPI samples is plotted in Fig. 3(b) against the effective number of layers. It is quite evident from Fig. 3(b), the DCP value is high and is almost constant for SPI WS_2_ nanostructures. Similar layer number independent DCP has been reported earlier in 3R stacked bulk MoS_2_ in which inversion symmetry is broken^23^. We believe that high DCP is due to the fact that in AB stacked SPI nanostructures, the inversion symmetry remains broken, similar to a ML.

In semiconductors, spin-dependent optical selection rules can give rise to circularly polarized luminescence due to the recombination of spin-polarized carriers excited by circularly polarized light^23^. The mechanism responsible for the robust circular polarization in TMDCs is not understood completely. In the case of WS_2_ BLs, high DCP has been proposed to be due to a combined effect of short exciton lifetime, small exciton binding energy, extra spin conserving channels and the coupling of spin-layer-valley degrees of freedom^13^. Using first-principles calculations, it was shown that high DCP could be explained by taking into account the hidden spin polarization of carriers that are localized on individual layers in a multilayer sample and it was predicted that the DCP would decrease monotonically as the number of layers increases^17^. However, our measurements show that in the case of SPI WS_2_ nanostructures, the DCP almost remains constant on increasing the number of layers. This suggests that the hidden spin polarization alone is not enough to explain the high DCP observed in WS_2_ nanostructures. In TMDCs, especially in tungsten dichalcogenides, the spin-orbit coupling strength is stronger than the interlayer hopping energy, which leads to the suppression of the interlayer scattering^15^. Because of the suppression of interlayer hopping and the spin-layer locking effect a high DCP has been reported earlier in BL WS_2_^13^. It has been shown that in 2H stacked MoS_2_ spin conserving intralayer/intervalley and interlayer/intravalley hopping leads to a reduction in the DCP with increasing number of layers, while the suppression of interlayer hopping in non-centrosymmetric 3R stacked MoS_2_ leads to a high DCP, irrespective of the number of layers^23^. We believe a similar suppression of interlayer/intravalley hopping is responsible for the high DCP, independent of the layer thickness observed in these AB stacked WS_2_ SPI nanostructures. Evidence for the reduction in interlayer/intravalley scattering is obtained from the PL studies using linearly polarized excitation described in the next section.

Based on TEM measurements, it has been proposed earlier that TMDC SPI nanostructures may exhibit a twisted AB stacking structure^19^. To check if our WS_2_ SPIs have a twisted structure, we carried out high-resolution TEM (HR-TEM) measurements. Supplementary Fig. S8 shows the HR-TEM images taken at two positions marked in Fig. S8(a), one taken on the top layer and the other taken on the bottom layer of the SPI nanostructure. The dashed arrows indicate the direction of the lattice sites. The dashed arrow in Fig. S8(b) was translated to Fig. S8(c) and shows that there is a twist angle of ~7 degrees between the top and bottom layer. It has been demonstrated earlier that such twisted structures exhibit strong non-linear effects due to broken symmetry^19^. We believe that the twisted nature of these WS_2_ SPI structures in which the inversion symmetry is already broken also helps to enhance the DCP. However, further studies are needed to understand how exactly the twisted nature affects the DCP.

### Linearly polarized excitation

The linearly polarized light can be considered as a coherent superposition of two circularly polarized components with opposite helicity, with a phase difference between them which determines the resulting polarization direction. Upon excitation with above band gap photons, the hot carriers quickly relax to the band edge with the emission of phonons. During this process, the phase information, which was transferred to the carriers during excitation is usually lost due to phonon scattering^15,32^. It has been shown that in WS_2_ BLs because of the suppression of interlayer scattering, the phase information is retained leading to significant linear polarization of the PL upon excitation with linearly polarized light^13,33,34^.

**Figure 4:**
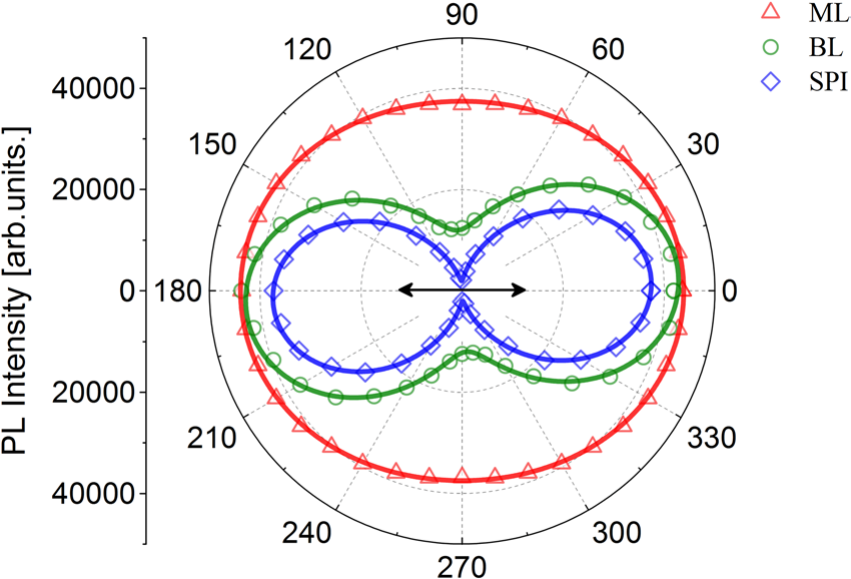
Polar plot of the PL intensity from WS_2_ samples at room temperature under near-resonant (633 nm) linearly polarized excitation. The double-headed arrow represents the direction of the excitation polarization.

We have studied the polarization of the PL under linear excitation. With linearly polarized, non-resonant excitation (532 nm), the PL from all the three kinds of nanostructures (ML, BL, and SPI) was randomly polarized. However, linearly polarized, near-resonant excitation (633 nm) of the BL and SPI nanostructures yielded PL with linear polarization characteristics. Figure 4 shows the PL intensity as a function of detection angle under linearly polarized near-resonant excitation of the WS_2_ samples at room temperature. As can be seen, the PL is predominantly polarized along the excitation polarization direction for BL and SPI samples. We defined the degree of linear polarization (DLP) as,

$${P}_{lin}( \% )=\frac{{I}_{\parallel }-{I}_{\perp }}{{I}_{\parallel }+{I}_{\perp }}\times 100$$

where ${I}_{\parallel }$ (*I*_⊥_) is the PL intensity parallel (perpendicular) to the excitation laser polarization. For near-resonant excitation, we observed DLP of ~7%, 56% and 89% for ML, BL and SPI samples, respectively. This further confirms that the interlayer/intravalley scattering is suppressed in WS_2_ BL and SPI samples under near-resonant excitation conditions leading to a very high degree of spin polarization.

In summary, TEM analysis of these WS_2_ SPI nanostructures show AB stacking order with broken inversion symmetry. Similar to the case of a ML, the absence of inversion symmetry results in high DCP from these SPI nanostructures, under near-resonant circularly polarized excitation. Linear polarization measurements show that interlayer/intravalley scattering is suppressed in these nanostructures. The suppression of interlayer/intravalley scattering in such SPI structures leads to a high DCP even at room temperature.

## Conclusions

We presented helicity resolved PL studies of WS_2_ spiral nanostructures carried out using non-resonant as well as near-resonant optical excitation. We report very high DCP ~94 ± 4% in WS_2_ SPI nanostructures excited using circularly polarized near-resonant (633 nm) light. TEM analysis showed that these SPI nanostructures have AB stacking in which the inversion symmetry is broken, and hence this leads to very high DCP. The dominant mechanism which leads to the depolarization of PL from TMDC MLs is the intervalley scattering due to acoustic phonons. A higher degree of polarization is obtained when using resonant excitation and at lower temperatures where the intervalley scattering is reduced. Analysis of the PL polarization and comparison of the PL spectra with that from WS_2_ MLs, suggests that in BL and multilayer SPI nanostructures the interlayer/intravalley scattering is further suppressed. The suppression of the interlayer/intravalley hopping also contributes to the high DCP in WS_2_ SPI nanostructures. This effect is more pronounced in tungsten dichalcogenides where the interlayer hopping strength is orders of magnitudes smaller than the spin-orbit splitting.

## Methods

### The growth of WS_2_ nanostructures

WO_3_ powder (Aldrich purses 99.9%) was dispersed in ethanol, and 10 μl of dispersion was drop-casted onto Si/SiO_2_ after sonicating for 30 min. The substrates were placed at the center of a single zone quartz tube reactor furnace (tube length 120 cm; 5 cm diameter; Thermo Scientific Lindberg Blue M), and 500 mg of Sulphur powder (Aldrich, purum 99.5%) was placed upstream towards the cooler side of the reactor. The quartz tube was evacuated for 30 min and refilled 3–4 times with ultra-high purity Argon gas to completely remove oxygen from the reaction zone. A steady flow of Argon at 200 sccm was maintained throughout the experiment. The furnace was heated to the reaction temperature. By this time, the temperature in the vicinity of sulphur powder reaches its evaporation temperature, and the sulphur fumes are carried towards the reaction zone by the Argon gas. After the deposition, the furnace is allowed to cool naturally. The morphology of the nanostructures was confirmed using AFM and TEM imaging.

### PL Measurements

PL measurements were carried out using a home built helicity-resolved set-up consisting of a Horiba-Jobin Yvon iHR320 spectrometer with a 300 g/mm grating, a 532 diode laser, a 633 nm He-Ne laser. The laser was focused on the sample using a short focal length lens (f = 13.86 mm), which gave an excitation spot size of ~5 microns diameter. The PL was collected using a long-working-distance 10x objective. Throughout the experiments, laser power was fixed to around ~0.1 mW. The experiment was performed at different temperatures in the range 100–300 K using a nitrogen flow cryostat. The schematic of the experimental setup is provided in Supplementary Fig. S9. The PL maps were obtained using a Raman microscope (XploRa PLUS, Horiba scientific), with a 532 nm laser. A 50x microscope objective gave a spot size of ~2–3 microns on the sample during PL mapping.

## Supplementary information

Supplementary Information
