# Supplementary material for: High degree of circular polarization in WS2 spiral nanostructures induced by broken symmetry
Source: Sci Rep. 2019 Feb 26;9:2784. doi: 10.1038/s41598-019-39246-7 (PMC6391472; doi:10.1038/s41598-019-39246-7)
Supplement: Supplementary file 1 — Supplementary Information [file 41598_2019_39246_MOESM1_ESM.pdf]

High degree of circular polarization in WS_2 spiral nanostructures induced by broken symmetry

Prahalad Kanti Barman, Prasad V. Sarma, M. M. Shaijumon and R. N. Kini*

School of Physics, Indian Institute of Science Education and Research Thiruvananthapuram (IISER-TVM), Maruthamala P.O., Vithura, Thiruvananthapuram, Kerala, 695551, India.

Email: rajeevkini@iisertvm.ac.in

Supplementary Information:

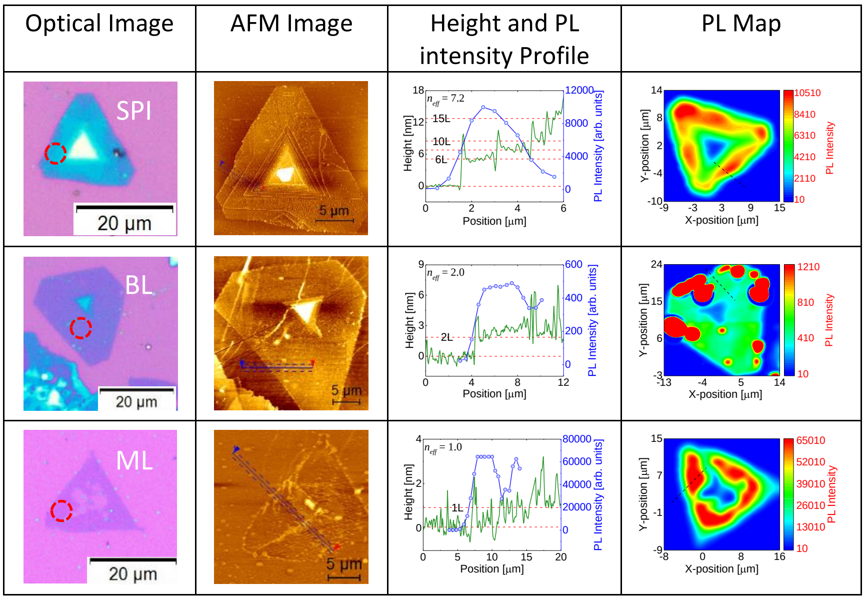

Figure S1. Optical microscope images, AFM images, PL maps and, the height and PL intensity profile of the layers, at the location on the samples indicated by the solid lines in the AFM images and PL maps, for WS_2 SPI, BL and ML samples.

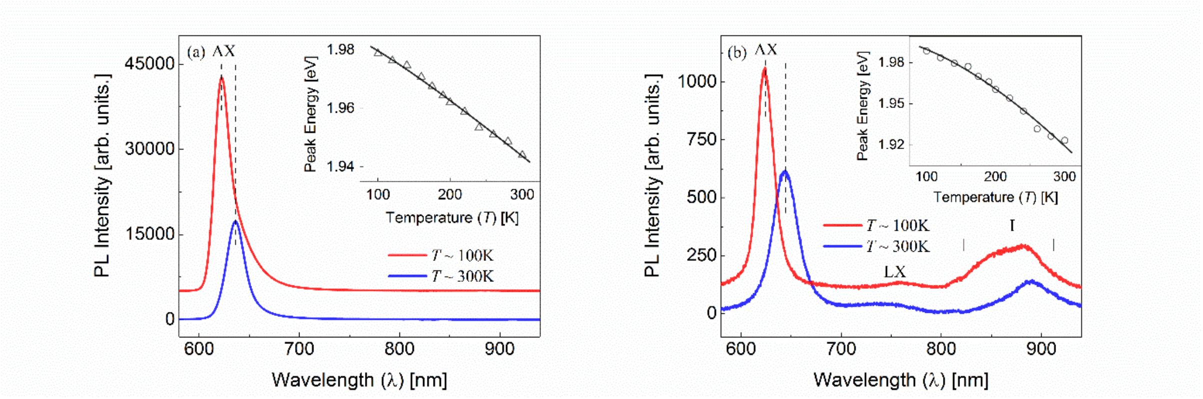

Figure S2. PL spectra of WS_2 (a) ML and (b) BL with 532 nm laser excitation at room temperature (~300 K) and low temperature (~100 K). Inset shows the AX peak energy as a function of temperature.

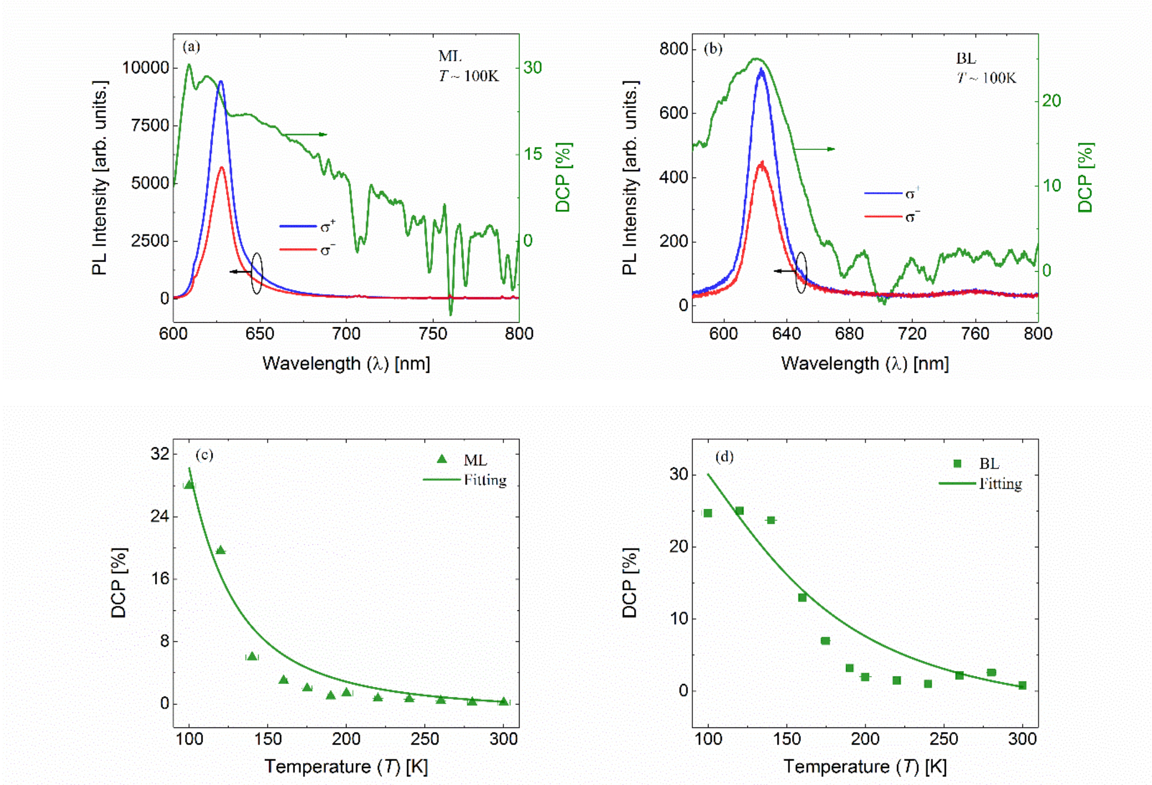

Figure S3. Helicity resolved PL spectra obtained using right handed circularly (σ^+) polarized 532 nm excitation and the corresponding DCP at ~100 K for WS_2 (a) ML and (b) BL samples. The DCP as a function of temperature for WS_2 (c) ML and (d) BL samples. The solid line is a fit to the experimental data as described in the main text.

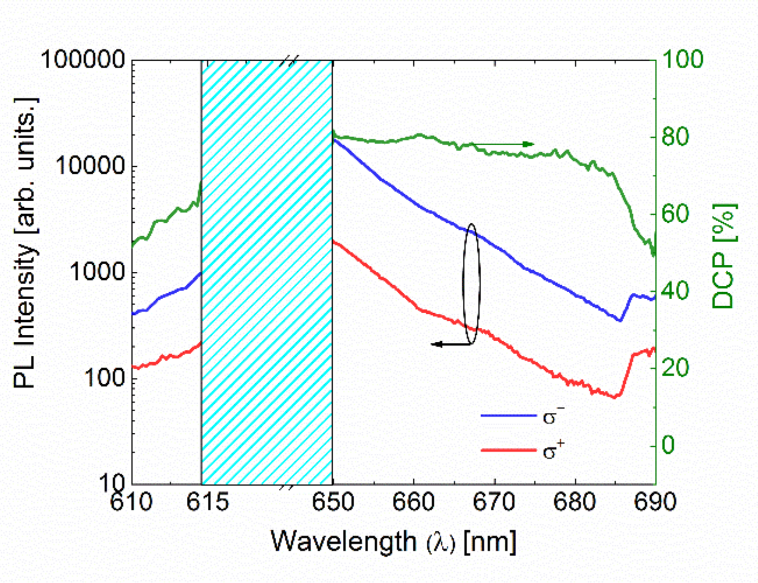

Figure S4. Helicity resolved PL spectra obtained by using right-handed circularly (σ^-) polarized near resonant (633 nm) excitation of twisted WS_2 SPI structure and the corresponding DCP at room temperature (~300 K). The shaded region represents the stop band of the notch filter used to block the laser light from reaching the detector. PL intensity is plotted on a logarithmic scale, in order to show that at around ~680 nm even though the intensity drops to less than 1/30th of the PL intensity at ~650 nm, the RCP and the LCP components of the PL still maintain a significant difference, giving rise to high DCP even away from the PL peak.

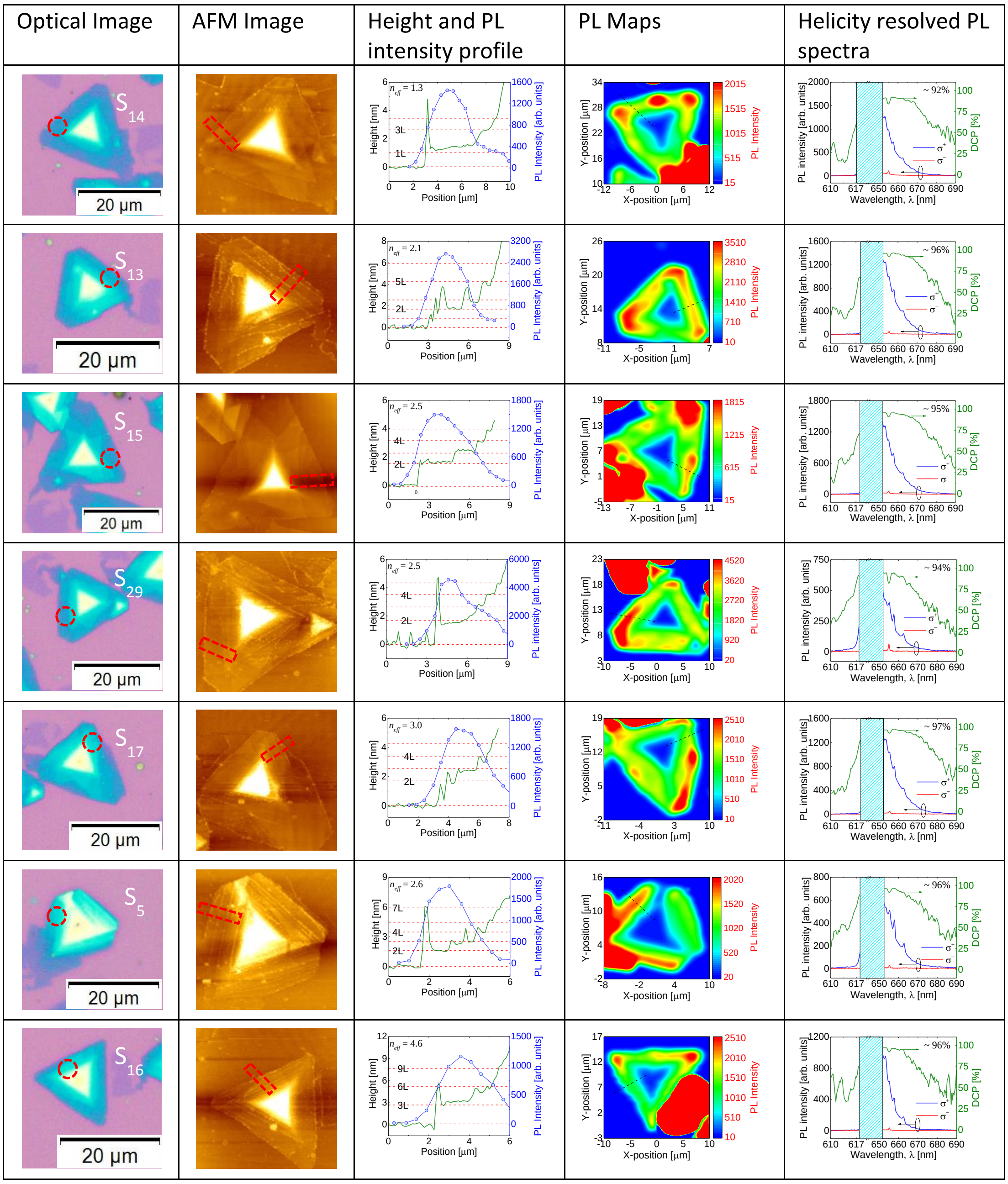

Figure S5. Optical microscope images, AFM images, PL maps and, the height and PL intensity profile of the layers, at the location on the samples indicated by the solid lines in the AFM images and PL maps, for several SPI WS_2 nano-structures.

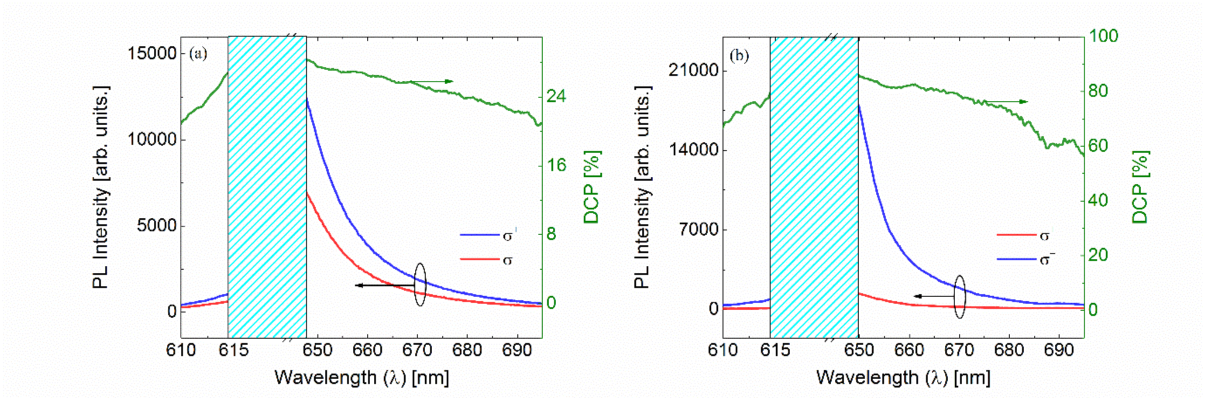

Figure S6. Helicity resolved PL spectra obtained by using right-handed circularly (σ^{+}) polarized near resonant (633 nm) excitation of WS_2 (a) ML and (b) BL samples and the corresponding DCP at room temperature (~300 K). The shaded region represents the stop band of the notch filter used to block the laser light from reaching the detector.

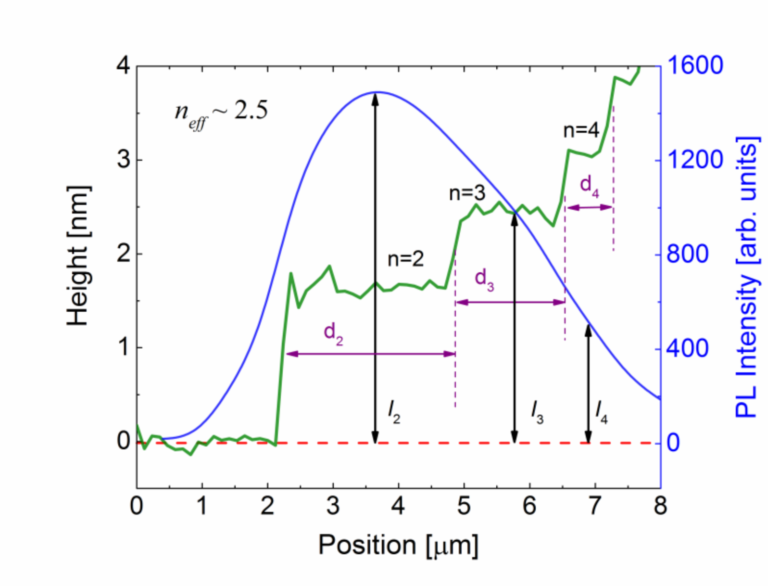

Figure S7. The height and PL intensity profile of one of the SPI samples, obtained from the AFM image and PL map. The effective layer number is obtained by taking a weighted average of the number of layers over the PL intensity as, $n_{eff} = \frac{\sum_n n I_n d_n}{\sum_n I_n d_n}$.

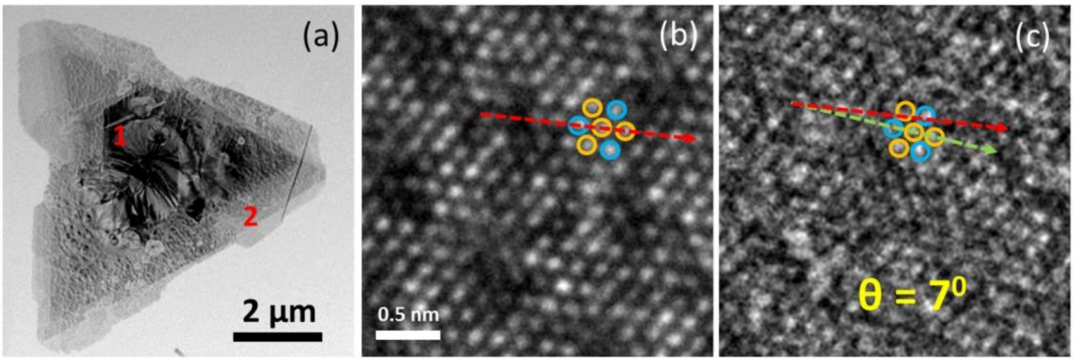

Figure S8. (a) HR-TEM image of SPI WS_2 domain. (b-c) The HRTEM images taken for top and bottom layer of SPI WS_2 respectively. Red and green dotted arrow line represents the line drawn through the direction of lattice sites. It was observed that top layer is rotated by an angle of ~ 7° compared with bottom layer.

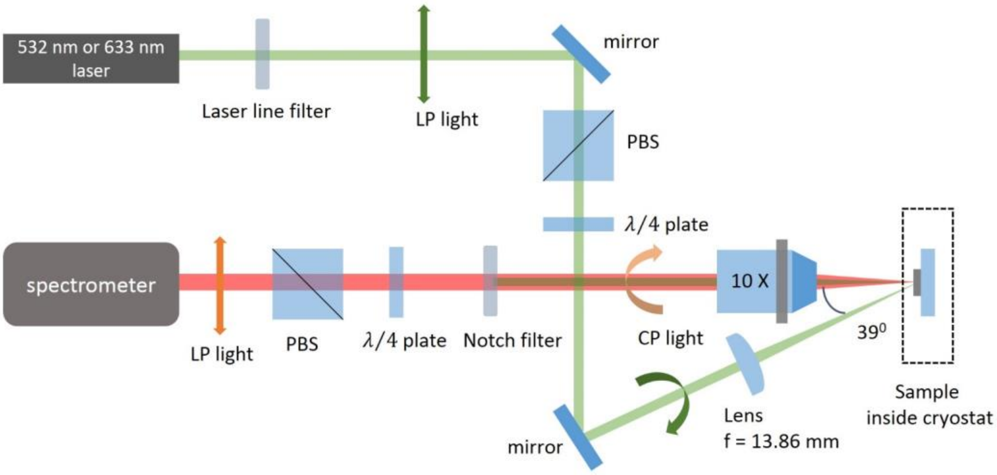

Figure S9. The schematic of the helicity resolved PL measurement setup. In this figure PBS refers to polarising beam-splitter, LP linearly polarized light, CP circularly (either LCP or RCP) polarized light, $(\lambda/4)$ quarter wave-plate.
